# Safety of intraoperative hypothermia for patients: meta-analyses of randomized controlled trials and observational studies

**DOI:** 10.1186/s12871-020-01065-z

**Published:** 2020-08-15

**Authors:** He Xu, Zijing Wang, Xin Guan, Yijuan Lu, Daniel Charles Malone, Jack Warren Salmon, Aixia Ma, Wenxi Tang

**Affiliations:** 1 School of International Pharmaceutical Business, China Pharmaceutical University, No.639 Longmian Street, Jiangning District, Nanjing, 211198 China; 2 Center for Pharmacoeconomics and Outcomes Research, China Pharmaceutical University, No.639 Longmian Street, Jiangning District, Nanjing, 211198 China; 3 College of Pharmacy, University of Utah, Salt Lake City, UT 84101 USA; 4 College of Pharmacy, University of Illinois Chicago, 833 South Wood Street, Chicago, IL 60612 USA

## Abstract

**Background:**

Previous studies have shown that intraoperative hypothermia was associated with higher risks of clinical adverse events, but we found otherwise from recent evidences. This study aims to synthesize the existing evidence evaluating safety of intraoperative hypothermia.

**Methods:**

Articles, reviews, ongoing trials and grey literatures were retrieved from PubMed, The Cochrane Library, Clinical Trails and CNKI (a Chinese national database) till February 2nd, 2019. Both randomized controlled trials and observational studies compared incidences of all sorts of intra- and post-operative consequences between hypothermia and normothermia were included. Researches comparing different warming systems were excluded. We also examined risks of hypothermia using lowered standards (35.5 °C and 35 °C) from a Chinese trial (ChiCTR-IPR-17011099).

**Results:**

A total of 9 RCT studies and 11 observational studies were included. RCT-synthesized results showed that intraoperative hypothermia was associated with higher risks of bleeding (MD = 131.90, 95%CI: 117.42, 146.38), surgical site infection (RD = 0.14, 95%CI: 0.06, 0.21) and shivering (RD = 0.32, 95%CI: 0.06, 0.58) but with no significant differences in duration of surgery, hospital stay or mortality. Observational study-synthesized evidences showed that intraoperative hypothermia did not result in higher risks in any of these adverse events. Results didn’t change even if the standard of hypothermia was lowered by 0.5–1.0 °C.

**Conclusions:**

The study indicates that the synthesized risks resulted by intra-operative hypothermia might be overestimated and the eligibility of 36 °C to define hypothermia is not sensitive enough. Given body-temperature protection has not been popularized in China, it is still critical to normalize the hypothermia prevention at this stage.

## Research in Context

### Evidence before this study

The safety and clinical effects of intraoperative warming have been extensively studied and reviewed. Individual studies have reported significantly increased risks of surgical site infection, blood loss, chills/shivering, and pain as well as a longer duration of surgery and longer stays in the post-anesthesia care unit (PACU) and hospital. However, no systematic comparison of postoperative outcomes in patients with as compared to without intraoperative hypothermia has been performed to date. Therefore, we searched the English and Chinese literature published before February 2019 to identify relevant research articles and registered clinical trials on this topic using four databases: Cochrane Library, PubMed, Clinical Trials (ClinicalTrials.gov), and China National Knowledge Infrastructure. The following search terms was used: 1) ((hypothermia) AND normothermia) AND (surgical site infection OR mortality OR blood loss OR pain OR chill OR shivering OR complications OR fluids infused OR duration of surgery OR duration of anesthesia OR duration of PACU OR length of stay OR readmission) AND (randomized controlled trial OR non-randomized OR non-randomised OR cohort OR observational OR investigation OR retrospective OR cross-sectional OR case control). Only randomized controlled trials (RCTs) or observational studies that included at least a hypothermic group and a normothermic group were included in the meta-analysis. Publications involving therapeutic hypothermia were excluded. All studies were evaluated with respect to bias and quality of conduct and reporting using Cochrane risk-of-bias tool or Observational Studies in Epidemiology (STROBE) statement

### Added value of this study

Intraoperative hypothermia is widely known operative risk that requires careful monitoring during surgical procedures. Using meta-analytical techniques, we provide evidence that the detrimental effects of intraoperative hypothermia are likely to be overestimated. Analysis of RCTs showed that the hypothermic group had significantly higher risks of surgical site infection, chills/shivering, and blood loss than did the normothermic group; however, no statistically significant difference was found in the duration of surgery, length of stay, or mortality. Furthermore, when the definition of intraoperative hypothermia was lowered to < 35·5 °C or < 35·0 °C, there was not reach statistical significance in the risk of other events except shivering. The difference between our findings and the current consensus is partly explained by the heterogeneity in the meta-analysis results, but it may also be attributable to the gaps in the causal chain from intraoperative hypothermia to adverse events, resulting in uncertainties, and to potential confounding effects that cannot be eliminated from RCTs (e.g., impact of other protective clinical practices that lead to reduced harm).

### Implications of all the available evidence

Under the current definition of intraoperative hypothermia as well as the tentatively lowered criteria for hypothermia, the evidence synthesized from the RCTs showed significantly higher intraoperative blood loss and incidences of surgical site infection and postoperative chills/shivering in the hypothermic than normothermic group. However, no significant differences were found in risks of other adverse events Furthermore, Evidence from observational studies also found no statistically significant difference in any postoperative adverse event. Therefore, the clinical harm reported in the studies evaluated herein appears lower than would be expected from the current consensus. These finding raises a question regarding the benefit of practices to prevent intraoperative hypothermia. Our findings are limited by the relatively small sample size and experimental designs. For more thorough assessment, future works should include larger-scale real-world studies and incorporate control over other medical practices (e.g., postoperative management that may offset the impact of intraoperative hypothermia).

## Background

Intraoperative hypothermia (core temperature of < 36 °C) is a common complication during surgery complications OR fluids infused OR duration of surgery OR [1]. Normal body temperature is maintained at approximately 37 °C by neurohumoral regulation to ensure stable physiological functions [2]. However, during surgery hypothermic events may occur as a result of multiple factors such as anesthesia, the operating room temperature, intraoperative warming practices, and infusions of fluids or blood product [3]. While many vital signs (e.g., blood pressure, heart rate, respiratory rate, and pulse) are routinely monitored during surgery, [4, 5] body temperature was commonly neglected until the past two decades, during which prevention of intraoperative hypothermia has become gradually accepted globally. Many organizations such as the American Society of Peri-Anesthesia Nurses, National Institute for Health and Care Excellence, Association of Peri-Operative Registered Nurses, and the Chinese Society of Anesthesia now recommend pre-warming before the operation, continuous intraoperative temperature monitoring and warming, and active warming in case of hypothermic events preoperatively or intraoperatively [3, 6–9].

Early studies of intraoperative hypothermia found the incidence ranged from 50 to 90% [10]. Improvements in the standardization of clinical practices and temperature-protective equipment has reduced this incidence. Recent studies have reported rates of 54% in distal gastrectomy [11], 37% in gastroenterological surgery [12], and 17% in hip fracture fixation [13]. An epidemiological survey conducted from 2014 to 2015 in China revealed an incidence of 44% [6]. The study found that patients who developed intraoperative hypothermia did not have a significantly increased risk of surgical site infection, a longer duration of intensive care unit (ICU) stay, or a higher 30-day mortality rate compared with patients who did not develop intraoperative hypothermia [6]. This unexpected finding was also supported by other studies. A randomized controlled trial (RCT) conducted from 2017 to 2018 in China (ChiCTR-IPR-17011099) showed a significantly lower incidence of intraoperative hypothermia in patients given active intraoperative warming than in patients who received regular passive warming during the operation [odds ratio (OR), 0.07; 95% confidence interval (CI), 0.04–0.14]; however, no significant difference was found in the incidences of intraoperative or postoperative adverse events [postoperative surgical site infection: OR = 1.11; 95% CI: 0.39–3.17; ICU admission: OR = 0.67; 95% CI: 0.38–1.21; postoperative blood loss: OR = 0.24; 95% CI: 0.03–2.14; duration of hospital stay: mean difference (MD) in days was − 1.25; 95% CI, − 6·15–4·31]. These findings raise questions regarding the impact of intraoperative hypothermia on clinical outcomes in the current clinical setting. Specifically, we asked whether such efforts translate into clinical benefits given the increasing development and use of intraoperative temperature protective techniques [14, 15].

The purpose of this study was to conduct a meta-analysis to synthesized evidence from published studies to assess the clinical harms of intraoperative hypothermia. Additionally, we explored the association between intraoperative hypothermia and the clinical harm, tested the differences in clinical injury under different hypothermia criteria, and discussed the possible factors underlying the lack of significance of hypothermia-induced harm.

## Methods

Data from RCTs and observational studies on the risks of adverse effects in patients with and without intraoperative hypothermia were identified and analyzed. Importantly, the findings were combined with data from a recently completed randomized controlled trial (RCT) to examine whether different hypothermia definition might alter the outcomes.

### Literature search

Evidence of studies related to surgical hypothermia was identified by searching four databases: Cochrane Library, PubMed, Clinical Trials (ClinicalTrials.gov), and China National Knowledge Infrastructure (CNKI). CNKI is currently the largest database of academic publications (e.g., research articles, dissertations, newspapers, conference proceedings, annals, and reference books) published in China. All information entered into the databases prior to February 2019 was included. References from identified studies were also evaluated for possible inclusion. In addition, results from an unpublished RCT is also included (see section 4, Methods).

Databases were searched using the keywords “intraoperative hypothermia” and “adverse events” in both English and Chinese. The resulting articles were reviewed to identify other potential search terms. The following keywords were commonly used in the articles: “surgical site infection,” “chill,” “shivering,” “complications,” “mortality,” “infusion,” “blood loss,” “pain,” “duration of surgery,” “duration of anesthesia,” “duration of PACU,” “length/days of stay,” and “readmission.”

New searches were conducted using the following terms: ((hypothermia) AND normothermia) AND (surgical site infection OR mortality OR blood loss OR pain OR chill OR shivering OR complications OR infusion OR duration of surgery OR duration of anesthesia OR duration of PACU OR length of stay OR readmission) AND (randomized controlled trial OR non-randomized OR non-randomised OR cohort OR observational OR investigation OR retrospective OR cross-sectional OR case control).

Studies meeting all of the following criteria were included: 1) inclusion of at least a hypothermic group and a normothermic group, 2) hypothermia defined as < 36 °C, and 3) reporting one or more of the following 12 adverse events: intraoperative blood loss/blood transfusion; surgical site infection; intraoperative or postoperative chills/shivering; complications; infusion; postoperative pain; duration of surgery; duration of anesthesia; duration of postanesthesia care unit (PACU) stay; duration/days of hospitalization; mortality; and readmission.

Studies meeting any of the following conditions were excluded: 1) inappropriate group division (e.g., grouping by use/non-use of warming practices with inadequate reporting of actual occurrence/absence of intraoperative hypothermia), 2) incomplete data (e.g., lack of standard deviation), 3) induction of hypothermia for treatment purposes (e.g., accidental cerebral injuries, myocardial conditions), 4) duplicate publication, 5) study reported in languages other than English or Chinese, or 6) unavailable full text.

### Information screening, retrieval, and quality assessment

The primary clinical harm of intraoperative hypothermia is the development of intraoperative and postoperative adverse events. Two researchers independently read all included studies for information screening, retrieval, and quality assessment. Disagreements were resolved by discussion or introduction of a third reviewer. The following items were retrieved: author name, year of publication, location where study was performed, population, study type, sample size, and outcomes. RCTs were assessed with the Cochrane risk-of-bias tool for quality of research methodology [16]. Observational studies were assessed with the Strengthening the Reporting of Observational Studies in Epidemiology (STROBE) statement [17], because of the lack of a universally accepted criterion on data quality, the studies were ranked according to the number of items reported and grouped around the median value to ensure that the sample was balanced.

### Statistical analysis

The risk difference (RD) and mean difference (MD) were used to calculate effect sizes. To determine if heterogeneity was present across the studies the Cochran Q test as estimated by the χ^2^ test (α = 0.05) and the I^2^ statistic (I^2^ ≥ 50%: substantial heterogeneity) was used. In case of minor heterogeneity (I^2^ < 50%), a fixed-effects model was used for the meta-analysis (α = 0.05). Otherwise, the source of the heterogeneity was further analyzed. If it was not possible to determine differences across the papers based on methodological and clinical factors, a random-effects model was used for the meta-analysis. When clinical or methodological factors contributed substantially to the heterogeneity, a subgroup analysis or sensitivity analysis was used or, alternatively, only qualitative description was performed. Statistical analysis was performed in Review Manager 5·3·5 (Cochrane Collaboration, www.cc-ims.net/RevMan).

### Impact of hypothermia definition on rates of adverse events

When intraoperative definition of hypothermia was evaluated < 36·0 °C, < 35·5 °C, or < 35·0 °C, the incidences of adverse events were analyzed from the data reported in a recently completed RCT (ChiCTR-IPR-17011099) in China and compared with the results from other studies. The RCT included 240 patients who underwent esophagectomy or pancreatectomy at Peking Union Medical College Hospital from 11 October 2016 to 28 March 2018. The patients were randomized to receive passive or active warming practices and were monitored for intraoperative hypothermia and adverse events (Table 1 shows the mean sample information). After the start of the operation, the temperatures of the eardrum and nasopharynx were measured every 30 min; if the temperature decreased to < 36 °C at any time during the operation, the patient was considered to have developed intraoperative hypothermia. Incidences calculated with the three tentative hypothermic definitions were analyzed with R 3.6.0 (α = 0.05) (R Foundation for Statistical Computing; www.r-project.org).

**Table 1:** Baseline characteristics of patients

	Experimental group (*n* = 122)	Control group (*n* = 118)	*P*-value^a^
Age	61·23 (9·79)	56·97 (11·23)	**0·002**
Sex			
Male	67·2% (82)	68·6% (81)	0·812
Female	32·8% (40)	31·4% (37)	
Ethnic group			
Han	92·6% (113)	94·1% (104)	0·654
Other	7·4% (9)	5·9% (7)	
Body mass/kg	66·05 (11·89)	65·45 (10·79)	0·963
Height/cm	167·80 (7·70))	167·68 (7·24)	0·870
Profession			
Regular employee	18·9% (23)	26·3% (31)	0·481
Short-time employee	0% (0)	0% (0)	
Part-time employee	0% (0)	0% (0)	
Self-employed	3·3% (4)	5·1% (6)	
Retiree	41·8% (51)	33·1% (39)	
Student	0% (0)	0·8% (1)	
Farmer	16·4% (20)	11·9% (14)	
Unemployed	19·7% (24)	22·9% (27)	
Medical insurance coverage			
Urban employee	41·8% (51)	49·2% (58)	0·098
Urban resident	7·4% (9)	5·9% (7)	
New rural cooperative	32·8% (40)	38·1% (45)	
Free medical care	6·6% (8)	1·7% (2)	
Commercial insurance	0% (0)	0% (0)	
Student insurance	0% (0)	0% (0)	
Other	5·7% (7)	1·7% (2)	
None	5·7% (7)	3·4% (4)	
Monthly income/RMB	3377·78 (1545·30)	3277·19 (1474·63)	0·617
Diabetes			
Yes	11·5% (14)	12·7% (15)	0·769
No	88·5% (108)	87·3% (103)	
Other diseases			
Yes	65·6% (80)	57·6% (68)	0·206
No	34·4% (42)	42·4% (50)	
Smoking			
Non-smoker	54·1% (66)	50·8% (60)	0·642
Quitter	17·2% (21)	18·6% (22)	
Smoker	28·7% (35)	30·5% (36)	
American Society of Anesthesiologists Classification			
I	11·5% (14)	22·9% (27)	0·059
II	82·0% (100)	70·3% (83)	
III	6·6% (8)	6·8% (8)	
Surgery			
Esophagectomy	52·5% (64)	47·5% (56)	0·439
Pancreatectomy	47·5% (58)	52·5% (62)	
Endoscopy			
Endoscopic surgery	19·7% (24)	20·3% (24)	0·979
Conversionof endoscopic surgery to thoractomy/laparotomy	15·6% (19)	14·4% (17)	
thoractomy/laparotomy	64·8% (79)	65·3% (77)	

^**a**^Chi-square test for categorical variables, T-test or rank sum test for continuous variables

Table 1 below describes the demographic characteristics of patients who were evaluated in a RCT examining the effects of passive or active warming.

## Results

**Fig. 1:**
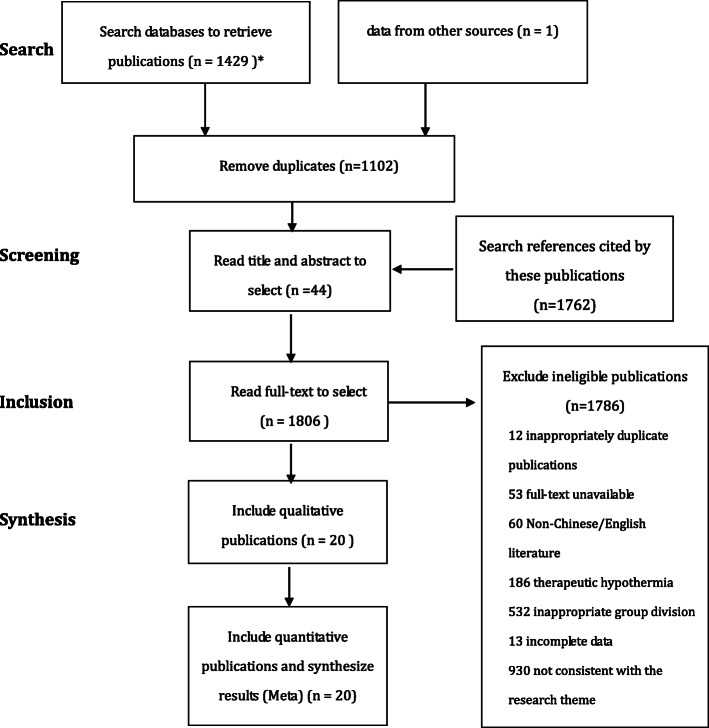
Flowchart illustrating screening of publications based on randomized controlled trials. *Databases and number of publications retrieved: PubMed (*n* = 753); The Cochrane Library (*n* = 507); CNKI(*n* = 165); clinical trial (*n* = 4)

The database search identified 614 publications RCTs and 818 related to observational studies [817 articles, 1 unpublished RCT (ChiCTR-IPR-17011099) after readjustment of grouping] related to surgical hypothermia. After applying inclusion and exclusion criteria, a total of 9 RCTs and 11 observational studies were included in the quantitative analysis (Fig. 1).

### Basic features of the included studies

The basic features included in the study are shown in Table 2.

**Table 2:** Basic features of the included studies

Studies	country	Population	Study type	Participants			Outcome indicators	Risk Difference (95%CI)	Mean Difference (95%CI)
				Hypothermic	Normothermic1	Normothermic2			
Yu et al (2010) [11]	China	patients undergoing opening radical resection of distal gastric cancer age:EG:60·20±13·30 CG:59·86±11·13	Randomised, controlled trial	32	54	\	Surgical site infection	0·15 [0·01, 0·29]	\
							Intraoperative bleed	\	158·48 [85·74, 231·22]
							Length of stay	\	1·99 [0·95, 3·03]
Zhang et al (2009) [18]	China	patients undergoing radical resection for carcinoma of esophagus age: EG:59±6 CG①: 59±7 CG②: 59±9^a^	Randomised, controlled trial	10	10^b^	10^b^	Shivering (EG vs CG1)	0·30 [-0·10, 0·70]	\
							Shivering (EG vs CG2)	0·60 [0·25, 0·95]	
Todd et al (2009) [19]	America	patients undergoing clipping of intracranial aneurysms after subarachnoid hemorrhage	Randomised, controlled trial	499	501	\	mortality	-0·01 [-0·04, 0·02]	\
Nathan et al (2004) [20]	Canada	patients undergoing elective coronary artery surgery with cardiopulmonary bypass aged over 60 years age:EG:68±6 CG:70±7	Randomised, controlled trial	71	73	\	Length of stay	\	-0·20 [-1·11, 0·71]
							mortality	0·03 [-0·02, 0·07]	\
Frank et al (1997) [21]	America	patients undergoing abdominal, thoracic, or vascular surgical procedures aged 71±1 years	Randomised, controlled trial	158	142	\	Intraoperative bleed	\	130·00 [115·16, 144·84]
							Length of surgery	\	-0·20 [-0·43, 0·03]
							Shivering	0·18 [0·10, 0·25]	\
							mortality	-0·00 [-0·03, 0·02]	\
Lenhardt et al (1997) [22]	America	patients undergoing elective major abdominal surgery age : EG:55±16 CG:56±17	Randomised, controlled trial	76	74	\	Length of surgery	-0·20 [-0·57, 0·17]	\
Kurz et al (2) (1996) [23]	America	patients undergoing elective colorectal resection age : EG:59±14 CG:61±15	Randomised, controlled trial	96	104	\	Surgical site infection	0·13 [0·04, 0·22]	\
							Length of stay	\	2·60 [1·05, 4·15]
Schmied et al (1996) [24]	Austria	patients undergoing unilateral total hip arthroplasties aged 63±10 year	Randomised, controlled trial	30	30	\	Intraoperative bleed	\	230·00 [64·89, 395·11]
Kurz et al (1996) [25]	America	patients undergoing elective colon surgery averagely aged 58 years age : EG:59±14 CG:57±15	Randomised, controlled trial	35	39	\	Length of surgery	\	0·20 [-0·35, 0·75]
Yamada et al (2019) [26]	Japan	patients undergoing orthopaedic surgery age : EG: 68·6 ±16·6 CG: 65·8 ±17·2	Observational study	1088	7833	\	Surgical site infection	-0·00[-0·01, 0·00]	\
							mortality	0·00 [-0·00, 0·01]	\
Xiehe (2018)^c^	China	Patients undergoing esophageal/pancreatic surgery age : EG: 60·6±9·6 CG: 58·0±11·4	Observational study	103	137	\	Surgical site infection	-0·00[-0·12, -0·00]	\
							Length of surgery	\	0·00 [-23·35, 23·35]
							Length of stay	\	-2·70 [-5·78, 0·38]
Williams et al (2018) [27]	England	patients undergoing total joint arthroplasty age : EG:72·0 ±10·0 CG: 71·3 ±10·3	Observational study	240	1815	\	Length of surgery	\	-5·00 [-9·08, -0·92]
							Length of stay	\	-0·50 [-0·92, -0·08]
							readmission	-0·01 [-0·04, 0·01]	\
Frisch et al(2) (2017) [28]	America	patients undergoing total hip and knee arthroplasty age : EG: 66·3±10·4 CG: 66·1±10·7	Observational study	887	1510	\	Length of surgery	\	0·70 [-2·05, 3·45]
							Length of stay	\	0·10 [-0·09, 0·29]
							readmission	0·00 [-0·02, 0·02]	\
Henriksen et al (2016) [29]	Denmark	patients diagnosed of infections age : EG: 75·8 [71·9-79·7] CCG: 72·8 [71·8-73·7]	Observational study	64	1216	\	mortality	0·19 [0·07, 0·31]	\
Frisch et al (2016) [13]	America	patients undergoing operative treatment of a hip fracture age : EG: 79·6±11·9 CG: 77·2±14·6	Observational study	260	1265	\	Length of surgery	\	-4·90 [-10·89, 1·09]
							Length of stay	\	-0·50 [-1·39, 0·39]
							readmission	-0·02 [-0·07, 0·03]	\
Tsuchida et al (2015) [12]	Japan	patients undergoing gastroenterologic surgery aged 15-92 years age : 61·2 ± 15·7 (15-92)	Observational study	528	881	\	Surgical site infection	-0·00[-0·04, 0·04]	\
Billeter et al (2014) [30]	America	patients undergoing elective operation age : EG: 61·3 ± 16·8 CG: 60·7 ± 16·3	Observational study	707	698	\	Surgical site infection	0·02 [-0·00, 0·04]	\
							Length of stay	\	5·50 [3·09, 7·91]
							mortality	0·13 [0·10, 0·16]	\
							Length of ICU stay	\	98·40 [60·75, 136·05]
Jeyadoss et al (2014) [31]	Australia	patients undergoing abdominal aortic aneurysm repair aged 71·8±6·9 years	Observational study	66	36	\	Length of stay	\	0·22 [-0·90, 1·34]
							Length of ICU stay	\	30·00 [11·11, 48·89]
Kebria et al (2012) [32]	England	patients undergoing debulking surgery aged 63·9± 11·7 years	Observational study	81	65	\	mortality	0·06 [0·00, 0·12]	\
							readmission	0·05 [-0·01, 0·11]	\
Williams et al (2) (2018) [33]	England	patients undergoing hip fracture operations age: EG: 87·1 ±7·8 CG: 84·7 ±7·8	Observational study	92	837	\	Length of surgery	\	-4·90 [-11·34, 1·54]
							Length of stay	\	0·30 [-1·19, 1·79]
							mortality	0·05 [-0·02, 0·11]	\
							Readmission	0·08 [0·01, 0·14]	\

^a^*EG* experimental group, *CG* control group

^b^This study actually used different warming methods as the control group, since the temperature of the control groups were below 36°C and the experimental group was above 36°C, they were all included· Experimental group warming method: active warming Thermacare○R; Control group 1 warming mode: after induction of general anesthesia, the lower body continued heating; Control group 2 warming mode: no warming·

^c^The RCT included 240 patients who underwent esophagectomy or pancreatectomy at Peking Union Medical College Hospital from 11 October 2016 to 28 March 2018. Identifier: ChiCTR-IPR-17011099. Available at: http://www.chictr.org.cn/historyversionpub.aspx?regno=ChiCTR-IPR-17011099

### Meta-analysis of adverse events

**Fig. 2:**
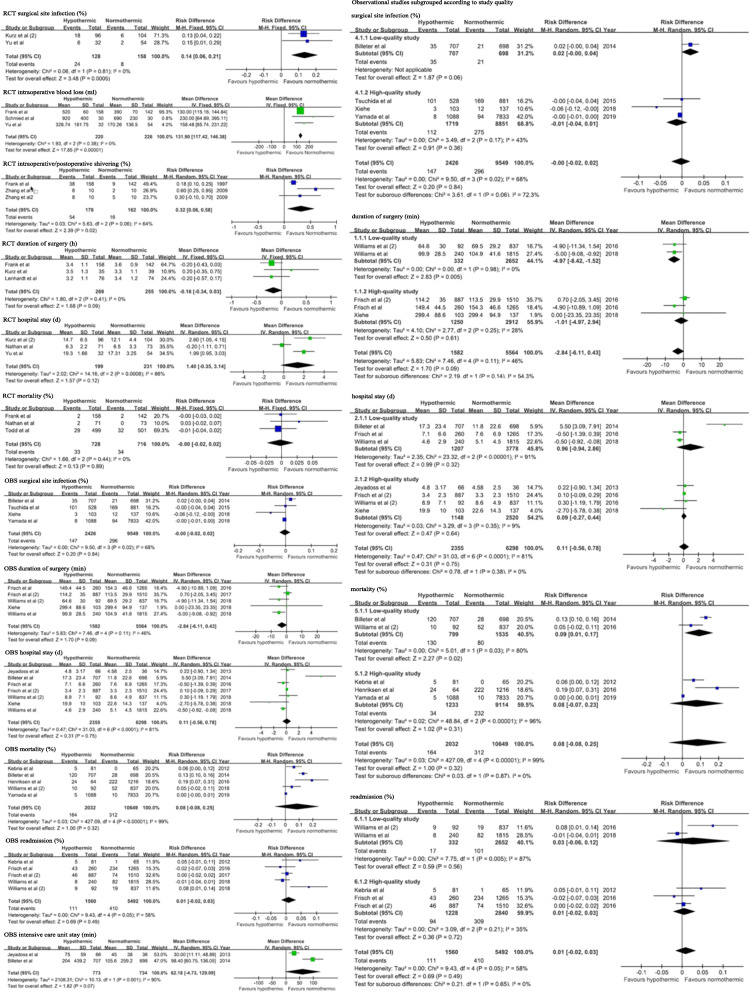
Meta-analysis of incidences of adverse events in hypothermic versus normothermic group

Figure 2 depicts the risks of adverse events in the hypothermic and normothermic groups. Results from the RCT studies, two adverse events (intraoperative/postoperative chills and length of stay) had substantial heterogeneity across the studies. Because only three RCTs were identified, a random-effects model was used instead of a subgroup analysis estimate the odds of these two outcomes. The meta-analysis of results reported that compared with the normothermic group, the hypothermic group had higher intraoperative blood loss (MD, 131.90 ml; 95% CI, 117.42–146.38) and higher incidences of surgical site infection (RD, 0.14; 95% CI, 0.06–0.21) and postoperative chills/shivering (RD, 0.32; 95% CI, 0.06–0.58). It should be note that the difference here point to the differences of statistical instead of a clinical one, which means whether the adverse events need to be treated, should be based on the actual situation The incidence of other adverse events (duration of surgery(h), length of stay(d), mortality) were not significantly different between the two groups. Results from the observational studies indicated the presence of heterogeneity; thus, they were analyzed by subgroup analyses according to the study quality. Studies of higher quality were observed to have lower heterogeneity, but no statistically significant differences in outcomes were detected.

### Assessment of risk of bias

**Fig. 3:**
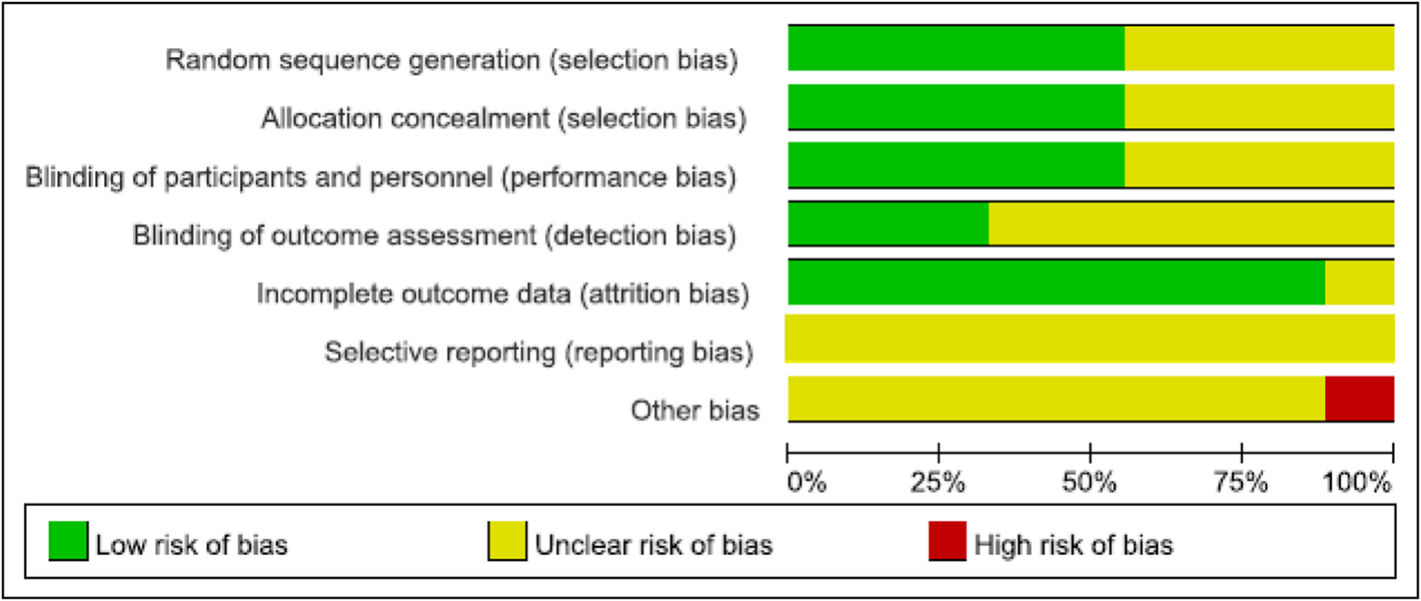
Assessment of risk of bias associated with randomized controlled trials

Figure 3 shows the results of the risk-of-bias assessment of the included RCTs. Of the nine RCTs, five described the method of generating the random sequence, and the other four contained inadequate information for confirming the validity of randomization. Five RCTs were double-blinded and described allocation concealment, and three used blinded outcome assessment. Eight RCTs included reports of complete outcomes. Among the nine RCTs, bias of selective reporting could be neither confirmed nor rejected because of inadequate information. One RCT reported the presence of other biases. Table 3 shows the results of the risk-of-bias assessment of the included observational studies. The studies of the highest and lowest qualities reported 86·36% and 54·54% of the items in the checklist, respectively (median, 68·18%), indicating generally satisfactory quality.

**Table 3:** Quality assessment of observational studies

Items	Yamada 2019	Williams 2018	Henriksen 2016	Frisch 2016	Williams2018	Frisch 2017	Tsuchida 2015	Billeter 2014	Jeyadoss 2013	Kebria 2012
Title and Abstract	√	√	√	√	√	√	√	√	√	√
Background/rationale	√	√	√	√	√	√	√	√	√	√
Objectives	√	√	√	√	√	√	√	√	√	×
Study design	√	√	√	√	√	√	√	√	√	√
Setting	√	√	√	√	×	√	√	×	√	√
Participants	√	×	√	√	√	√	√	√	√	√
Variables	×	×	×	×	×	×	×	×	×	×
Data sources / measurement	√	×	√	√	×	√	√	√	√	√
Bias	√	×	×	×	×	×	×	×	×	×
Study size	×	×	×	×	×	×	×	×	×	×
Quantitative variables	√	√	√	√	√	√	√	√	√	√
Statistical methods	×	×	×	×	×	×	×	×	×	×
Participants	√	×	√	√	√	√	√	×	√	√
Descriptive data	√	×	√	√	×	×	×	×	×	×
Outcome data	√	√	√	√	√	√	√	√	√	√
Main results	√	×	√	×	×	√	×	×	×	×
Other analyses	√	√	×	×	×	√	√	√	√	×
Key results	√	√	√	√	√	√	√	√	√	√
Limitations	√	√	√	√	√	√	√	√	√	×
Interpretation	√	√	√	√	√	√	√	√	√	√
Generalisability	√	√	√	√	√	√	×	√	√	√
Funding	√	×	√	×	×	×	√	×	×	×
Percentage	86·36%	54·55%	77·27%	68·18%	54·55%	72·72%	68·18%	59·09%	68·18%	54·54%
Median	68·18%									

### Effects of hypothermia definition on occurrence of adverse events

Because the type of surgery affects clinical outcomes, the included studies were sub-grouped by the type of surgery and then analyzed assuming three definitions of intraoperative hypothermia: < 36.0 °C, < 35.5 °C, or < 35.0 °C (Table 4). The results showed that only when intraoperative hypothermia was defined as < 35.0 °C was the incidence of chills significantly higher (*p* < 0.05) in the hypothermic group undergoing esophagectomy than in the normothermic group.

**Table 4:** Incidences of adverse events under intraoperative hypothermia definition of <36·0°C, <35·5°C, or <35·0°C

	36·0°C			35·5°			35·0°C		
	Normothermia	Hypothermia	***P***^**a**^	Normothermia	Hypothermia	***P***^**a**^	Normothermia	Hypothermia	***P***^**a**^
Esophagectomy									
Intraoperative bleed ml	285·19 (171·11)	255·70 (144·24)	0·297	273·29 (157·37)	263·28 (160·52)	0·511	279·24 (162·95)	213·66 (109·77)	0·108
Length of stay d	20·95 (16·40)	21·60 (36·73)	0·277	20·44 (14·97)	22·95 (45·23)	0·066	22·10±(30·76)	16·29 (6·32)	0·140
Length of PACU min	0·43 (0·09)	0·39 (0·14)	0·302	0·37 (0·16)	0·38 (0·16)	0·868	0·38 (0·17)	0·37 (0·10)	0·478
Superficial Surgical Site Infection %	2/58	0/62	0·232	2/80	0/40	0·552	2/103	0/17	1·000
Shivering %	0/58	2/62	0·496	0/80	2/40	0·109	**0/103**	**2/17**	**0·019**
Pancreatectomy									
Intraoperative bleed ml	541·48 (434·55)	542·71 (506·23)	0·808	528·87 (416·52)	615·73 (154·90)	0·977	527·12 (421·15)	1118·33 (1300·47)	0·528
Length of stay d	23·82 (12·59)	24·22 (11·75)	0·844	23·20 (11·77)	27·50 (14·42)	0·273	23·75 (12·26)	27·33 (12·58)	0·533
Length of PACU min	0·46 (0·18)	0·50 (0·18)	0·272	0·46 (0·19)	0·51 (0·25)	0·166	0·46 (0·20)	0·33	0·337
Superficial Surgical Site Infection %	2/79	1/41	1·000	2/102	1/18	0·389	3/117	0/3	1·000
Shivering %	0/79	0/41	-	0/102	0/18	-	0/117	0/3	-

^a^Chi-square test for categorical variables, T-test or rank sum test for continuous variables

## Discussion

### Clinical harm of intraoperative hypothermia is lower than expected

This analysis suggests that the risks of intraoperative hypothermia-associated adverse events are lower than the current consensus and other reports [19, 20, 26]. The difference may be partly attributable to when the studies were conducted and perhaps substantial variation in the type and duration of surgery. Other factors may have also contributed, such as the long causal chain from intraoperative to adverse events (e.g. mortality) and the confounding effects of medical practice. Surgical site infections are a key indicator of the quality of medical service [34]; therefore, surgeons may tend to be conservative in interpreting whether an infection is present, leading to a lower reported incidence in more modern times as compared to earlier decades.

Both RCTs and observational studies reported the incidence of surgical infection, duration of surgery, duration of hospital stay, and mortality rate. Additionally, RCTs were more likely to have reported intraoperative blood loss and chills/shivering, whereas the observational studies focused on readmission rates and duration of ICU stays. The meta-analysis of RCTs found there was significantly higher intraoperative blood loss, surgical site infection, and intraoperative/postoperative chills/shivering in patients experiencing hypothermia than those who did not. In comparison, the high-quality observational studies showed no statistically significant differences in the incidences of adverse events between the two groups. The different findings from these two categories of studies in the present meta-analysis may be explained by their different experimental designs, patient characteristics, and types of surgery.

### Is 36·0 °C an adequately sensitive criterion for intraoperative hypothermia?

Our analysis was based on the current consensus of the definition of intraoperative hypothermia (< 36·0 °C) and found fewer differences than other studies. This raised the question of whether < 36·0 °C is an adequately sensitive cut point for classifying intraoperative body temperature as normal or hypothermic.

Our exploratory analyses showed persistent gaps between the expected and actual incidences of adverse events. Even assuming a tentative definition of intraoperative hypothermia of < 35·0 °C, except for chills the clinical risks of adverse events in the hypothermic group were not significantly higher than those in the normothermic group. This is partly explained by the unequal sizes of the two groups; however, it may also be related to the intrinsically moderate harm of intraoperative hypothermia. Several earlier studies have shown similar results. In a recent registry study of 7908 colorectal patients, Sessler et al. founded that intraoperative core temperature < 35.5 °C was associated with an increased odds ratio of serious infection, but superficial infection and duration of hospitalization were not significantly related to intraoperative core temperature. In the subgroup analysis of colorectal cancer patients, none of the above was related to intraoperative core temperature [35]. In 2007, Iwata et al. reported that when intraoperative hypothermia was defined as a body temperature of < 35·5 °C, the durations of surgery in the hypothermic and normothermic groups were not significantly different (396 ± 204 vs. 327 ± 97 min) [36]. Two other studies showed that when intraoperative hypothermia was defined as < 35·0 °C, the hypothermic group had a significantly longer duration of surgery than the normothermic group (396 ± 204 vs. 252 ± 180 min) [37] and a non-significantly higher mortality rate [12·5% (5/40) vs. 4·5% (3/67)] [38]. These data also support our finding that in current clinical practice, the harm induced by intraoperative hypothermia is less than previously established.

### Do we still need to prevent intraoperative hypothermia?

The awareness of the potential for harm associated with intraoperative hypothermia has been increasing since the 1990s, with an increased focus on preventing hypothermia. However, the present study suggests that the current consensus on its harm is likely overestimated. Notably, this does not imply that the severe adverse effects of intraoperative hypothermia and its risk can be ignored. In fact, practices to prevent intraoperative hypothermia are often initiated.

Currently, perioperative body temperature monitoring is not routinely performed in China, with passive measures used to prevent hypothermia [1]. Yi et al. reported that in China, only 10·7% of patients received active temperature monitoring and treatment and that the use of more economically efficient equipment was unpopular [39]. Nevertheless, economic factors should not be the principal consideration in prevention of intraoperative hypothermia. Instead, the focus should be aimed at improving the awareness of protecting body temperature as a critical life sign. Additionally, a full picture of the clinical and economic benefits of hypothermia prevention needs to be revealed under various definitions of hypothermia.

### Research novelty and limitations

This meta-analysis addressed the harm of intraoperative hypothermia based on synthesizing evidence from multiple studies and thus overcomes the limitations from examining individual studies. The findings of this study provide new insight into the clinical value of intraoperative hypothermia and its prevention. We also examined the possibility of refining the current definition of intraoperative hypothermia. Evidence from multiple sources (analyses of data from studies adopting a lower temperature criterion, a recently completed RCT in China, and meta-analysis of publications) suggest that differences exist from pre-conceived notions of adverse events due the surgical hypothermia.

The publications were categorized into RCTs and observational studies for separate meta-analyses, and the different findings of the two categories may be primarily attributed to heterogeneity within individual studies (e.g., surgery, procedures, and location of study) rather than the methodological design of the current meta-analysis (i.e., RCT vs. observational). Whether data from RCTs and observational studies can be directly combined for one analysis remains debatable [40, 41]; therefore, we separated them. We have tried to compare the heterogeneity of RCTs and observational studies as a whole and subgroups, and found that separating them can effectively reduce the heterogeneity. Because of the relatively small number of studies included, a subgroup analysis (e.g., by the type of surgery) is methodologically questionable. Therefore, we only performed analyses for uncertainty using an effects model and study quality, which may have affected the robustness of the meta-analysis findings.

## Conclusions

The evidence from our research suggests that differences exist from pre-conceived notions of adverse events due the surgical hypothermia, and 36·0 °C is not an adequately sensitive criterion for intraoperative hypothermia. These findings may contribute to a better understanding of intraoperative hypothermia and its prevention in clinical practice in fields such as anesthesiology and nursing. For more thorough assessment, future works should include larger-scale real-world studies and incorporate control over other medical practices.

## Data Availability

The data that support the findings of this study are available from Peking Union Medical College Hospital, but restrictions apply to the availability of these data, which were used under license for the current study, and so are not publicly available. Data are however available from the authors upon reasonable request and with permission of Peking Union Medical College Hospital.

## Abbreviations

CI: Confidence interval
ICU: Intensive care unit
MD: Mean difference
OR: Odds ratio
PACU: Post anesthesia care unit
RCT: Randomized controlled trial
STROBE: Strengthening the Reporting of Observational Studies in Epidemiology

## References

[1] Burns SM, Piotrowski K, Caraffa G (2010). Incidence of Postoperative Hypothermia and the Relationship to Clinical Variables. J PeriAnesth Nurs.

[2] Hart SR, Bordes B, Hart J (2011). Unintended perioperative hypothermia. Ochsner J.

[3] Ma ZL, Yi J (2017). Expert consensus on prevention and treatment of hypothermia in perioperative patients (2017). Med J Peking Union Med Coll Hosp.

[4] Chen XL, Yu RL, Chen LR (2009). Application of personalized nursing in perioperative period. J Pract Med.

[5] Xue Li F, Li SL, Liang LG (2001). The value of medical thorascopy. Chin J Tuberc Respir Dis.

[6] Yi J, Lei Y, Xu S (2017). Intraoperative hypothermia and its clinical outcomes in patients undergoing general anesthesia: National study in China. PLoS One.

[7] Hooper VD, Chard R, Clifford T (2010). ASPAN’s Evidence-Based Clinical Practice Guideline for the Promotion of Perioperative Normothermia: Second Edition. J PeriAnesth Nurs.

[8] Burger L, Fitzpatrick J (2009). Prevention of inadvertent perioperative hypothermia. Br J Nurs.

[9] NICE (2016). Hypothermia: prevention and management in adults having surgery.

[10] Knaepel A (2012). Inadvertent perioperative hypothermia: a literature review. J Perioperative Pract.

[11] Yu ZH. Clinical and basic researches of intraoperative hypothermia. Dalian Med Uni Gen Sur. 2010.

[12] Tsuchida T, Takesue Y, Ichiki K (2016). Influence of Peri-Operative Hypothermia on Surgical Site Infection in Prolonged Gastroenterological Surgery. Surg Infect.

[13] Frisch NB, Pepper AM, Jildeh TR (2016). Intraoperative Hypothermia During Surgical Fixation of Hip Fractures. Orthopedics.

[14] John M, Crook D, Dasari K (2016). Comparison of resistive heating and forced-air warming to prevent inadvertent perioperative hypothermia. Br J Anaesth.

[15] Kim E, Lee S, Lim Y (2015). Effect of a new heated and humidified breathing circuit with a fluid-warming device on intraoperative core temperature: a prospective randomized study. J Anesth.

[16] Higgins JPT, Altman DG, Gotzsche PC (2011). The Cochrane Collaboration's tool for assessing risk of bias in randomised trials. BMJ.

[17] von Elm E, Altman DG, Egger M (2014). The Strengthening the Reporting of Observational Studies in Epidemiology (STROBE) Statement: Guidelines for reporting observational studies. Int J Surg.

[18] Zhang J, Jing C (2009). Outcomes of two temperature maintenance strategies during radical resection for carcinoma of oesophagus and their effects on postoperative shivering. J Shanghai Jiaotong Univ (Med Sci).

[19] Todd MM, Hindman BJ, Clarke WR (2009). Perioperative fever and outcome in surgical patients with aneurysmal subarachnoid hemorrhage. Neurosurgery.

[20] Nathan HJ, Parlea L, Dupuis J (2004). Safety of deliberate intraoperative and postoperative hypothermia for patients undergoing coronary artery surgery: A randomized trial. J Thorac Cardiovasc Surg.

[21] Frank SM, Fleisher LA, Breslow MJ (1997). Perioperative maintenance of normothermia reduces the incidence of morbid cardiac events. A randomized clinical trial. JAMA.

[22] Lenhardt R, Marker E, Goll V (1997). Mild intraoperative hypothermia prolongs postanesthetic recovery. Anesthesiology.

[23] Kurz A, Sessler DI, Enhardt R (1996). Perioperative normothermia to reduce the incidence of surgical-wound infection and shorten hospitalization. N Engl J Med.

[24] Schmied H, Kurz A, Sessler DI (1996). Mild hypothermia increases blood loss and transfusion requirements during total hip arthroplasty. Lancet.

[25] Kurz A, Sessler DI, Narzt E (1995). Postoperative hemodynamic and thermoregulatory consequences of intraoperative core hypothermia. J Clin Anesth.

[26] Yamada K, Nakajima K, Nakamoto H, et al. Association between Normothermia at the End of Surgery and Postoperative Complications following Orthopaedic Surgery. Clin Infect Dis. 2019. 10.1093/cid/ciz213 30863863

[27] Williams M, El-Houdiri Y (2018). Inadvertent hypothermia in hip and knee total joint arthroplasty. J Orthop.

[28] Frisch NB, Pepper AM, Rooney E (2017). Intraoperative Hypothermia in Total Hip and Knee Arthroplasty. Orthopedics.

[29] Henriksen DP, Havshøj U, Pedersen PB (2016). Hospitalized acute patients with fever and severe infection have lower mortality than patients with hypo- or normothermia: a follow-up study. QJM.

[30] Billeter AT, Hohmann SF, Druen D (2014). Unintentional perioperative hypothermia is associated with severe complications and high mortality in elective operations. Surgery.

[31] Jeyadoss J, Thiruvenkatarajan V, Watts RW (2013). Intraoperative hypothermia is associated with an increased intensive care unit length-of-stay in patients undergoing elective open abdominal aortic aneurysm surgery: a retrospective cohort study. Anaesth Intensive Care.

[32] Moslemi-Kebria M, El-Nashar SA, Aletti GD (2012). Intraoperative hypothermia during cytoreductive surgery for ovarian cancer and perioperative morbidity. Obstet Gynecol.

[33] Williams M, Ng M, Ashworth M (2018). What is the incidence of inadvertent hypothermia in elderly hip fracture patients and is this associated with increased readmissions and mortality?. J Orthop.

[34] Wang HQ, Wang W, Yan F (2018). Comparative analysis on international health care quality evaluation indicator systems. Chin Health Resour.

[35] Walters MJ, Tanios M, Koyuncu O (2020). Intraoperative core temperature and infectious complications after colorectal surgery: A registry analysis. J Clin Anesth.

[36] Iwata Y, Newburger JW, Zurakowski D (2007). Postoperative Hypothermia and Blood Loss After the Neonatal Arterial Switch Procedure. Ann Thorac Surg.

[37] Coon D, Michaels J, Gusenoff JA (2012). Hypothermia and Complications in Postbariatric Body Contouring. Plast Reconstr Surg.

[38] Sun Y, Jia L, Yu W, et al. The changes of intraoperative body temperature in adult liver transplantation: A retrospective study. Hepatobiliary Pancreat Dis Int. 2018;17(6):496–501. 10.1016/j.hbpd.2018.08.006 30205926

[39] Yi J, Xiang Z, Deng X, et al. Incidence of inadvertent intraoperative hypothermia and its risk factors in patients undergoing general anesthesia in Beijing: a prospective regional survey. PLoS One. 2015;10(9):e136136. 10.1371/journal.pone.0136136 PMC4567074 26360773

[40] Benson K, Hartz J (2000). A comparison of observational studies and randomized, controlled trials. N Engl J Med.

[41] Britton A, Mckee M (1998). Choosing between randomised and non-randomised studies: a systematic review. Health Technol Assess.

